# Data on draft genome sequence of *Bacillus* sp. strain VKPM B-3276 isolated from *Culex pipiens* larvae

**DOI:** 10.1016/j.dib.2019.103757

**Published:** 2019-03-07

**Authors:** V.V. Zinina, A.A. Korzhenkov, A.V. Tepliuk, A.A. Kanikovskaja, M.V. Patrushev, I.V. Kublanov, S.V. Toshchakov

**Affiliations:** a National Research Center “Kurchatov Institute”, Moscow, 123182, Russia; b Federal Institution “State Research Institute of Genetics and Selection of Industrial Microorganisms” of the National Research Center “Kurchatov Institute”, Moscow, 117545, Russia

**Keywords:** Draft genome assembly, *De novo* assembly, Entomopathogenic bacteria, *Bacillus*, Mosquito biocontrol

## Abstract

The draft genome sequence of *Bacillus* sp. VKPM B-3276, a mesophilic, gram-positive bacterium, isolated from dead *Culex pipiens* larvae is presented. This strain was deposited in the Russian National Collection of Industrial Microorganisms as a prospective candidate for development of new entomopathogenic agents. The genome of *Bacillus* sp. VKPM B-3276 was 6,126,346 bp in length with predicted completeness of 99.43%. Genome analysis identified 6518 protein-coding sequences and 111 RNAs genes. 13% (271) of the protein-coding genes were assigned to “Carbohydrates” subsystem category, according to RAST/SEED. Among them about 50 enzymes, responsible for chitin, peptidoglycan and related molecules decomposition, were found. The draft genome of strain VKPM B-3276 was deposited at DBJ/EMBL/GenBank under the accession nos. RZHM00000000, PRJNA511803 and SAMN10644103 for Genome, Bioproject and Biosample, respectively.

**Table:** Specifications table.

Subject area	Biology, Microbiology
More specific subject area	Microbial biotechnology
Type of data	Genomic sequence, predicted genes and annotation of respective proteins, deposited in NCBI database and available by links provided within article; heatmap of average nucleotide identity between type strain genome assemblies of “*Bacilluscereus* group”, and histogram of genes involved in degradation of chitin, peptidoglycan and related compounds presented within an article;
How data was acquired	*De novo* whole genome sequencing with Illumina MiSeq
Data format	Analyzed and annotated draft genome assembly
Experimental factors	Extraction of genomic DNA from a pure culture, fragment library preparation, Illumina sequencing, *de novo* assembly and annotation procedures
Experimental features	Extraction of genomic DNA was performed with standard phenol-chloroform method; fragment library was prepared with KAPA HyperPlus™ Kit; sequencing was performed with Illumina MiSeq™ system. The genome was assembled using SPAdes and annotated with RAST web server
Data source location	The culture of strain VKPM B-3276 is deposited in Russian National Collection of Industrial Microorganisms (VKPM) in Moscow, Russian Federation. http://vkpm.genetika.ru/katalog-mikroorganizmov/show21240/
Data accessibility	Data are publicly available at NCBI Genbank. The Biosample, Bioproject and assembly/WGS accession numbers are: SAMN10644103 (https://www.ncbi.nlm.nih.gov/biosample/SAMN10644103/) PRJNA511803 (https://www.ncbi.nlm.nih.gov/bioproject/PRJNA511806) and RZHM00000000 (https://www.ncbi.nlm.nih.gov/nuccore/RZHM00000000), respectively.

**Table:** 

**Value of the data** • This particular *Bacillus* sp. strain VKPM B-3276 was isolated from *Culex pipiens* larvae and showed significant entomopathogenic activity [2], therefore could be regarded as prospective entomocide. • The genome encodes a high number of various enzymes, participating in chitin and peptidoglycan degradation, which could be relevant in medicine (antimicrobial agents) or for waste utilization (chitin bioconversion). • According to whole genome alignment data *Bacillus* sp. strain VKPM B-3276 may be regarded as a new subspecies inside “*Bacillus cereus* group”. • Data on genome sequence of strain VKPM B-3276 can be used to search and characterize novel biotechnology-relevant enzymes and gene clusters.

## 1 Data

**Fig. 1:**
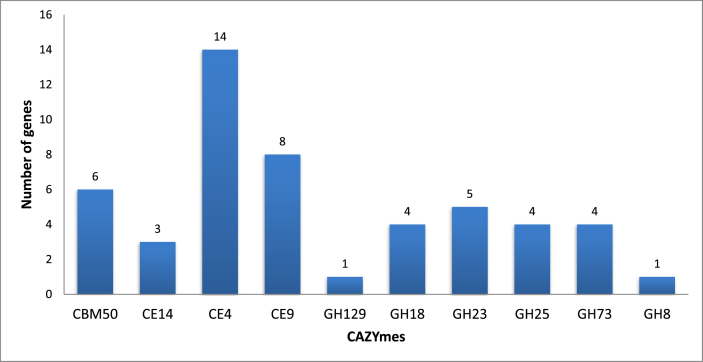
Genes, encoding proteins involved in degradation of chitin, peptidoglycan and related compounds. CBM50 - CBM module of enzymes, cleaving either chitin or peptidoglycan; CE14 - diacetylchitobiose deacetylase, putative; CE4 - chitin deacetylase, putative; CE9 - *N*-acetylglucosamine 6-phosphate deacetylase; GH129 - α-*N*-acetylgalactosaminidase; GH18 - chitinase, putative; GH23 - lysozyme, putative; GH25 - lysozyme, putative; GH73 - lysozyme, putative; GH8 - сhitosanase.

Bacillus sp. strain VKPM B-3276 was isolated from *Culex pipiens* larvae as an entomopathogenic agent [1]. Its genome was sequenced using Illumina Miseq platform to identify genes, responsible for its entomopathogenic properties. *De novo* assembly resulted in 176 contigs with average coverage of 44x. Total length of the assembly was equal to 6,126,346  bp with a G + C content of 35%. Automatic annotation by RAST (Rapid Annotation using Subsystems Technology) server [2] identified 6518 protein-coding and 111 RNA genes. Protein-coding sequences were organized in 358 subsystems, among which the most numerous were “Amino acids and derivatives” (395 genes), “Carbohydrates” (271), “Protein” (191) and “Cofactors, Vitamins, Prosthetic Groups, Pigments” (187). From almost 200 Carbohydrate Active enZymes (CAZymes) [3], detected using the dbCAN server [4] 50 were predicted to participate in decomposition of chitin and peptidoglycan and their derivatives (Fig. 1). The latter is well correlated with the isolation source of this strain as well as its entomocidic capabilities [1]. According to sequence comparisons, some of these enzymes are only distantly related to currently known members of CAZyme families and/or representing recently proposed families with limited number of members. E.g. VKPM B-3276 genome possesses a gene for GH129, a family, for which the only characterized member - α-*N*-acetylgalactosaminidase, possibly involved in mucin degradation [5]. This observation emphasizes the potential of this strain for other than insecticide-related applications.

Analysis of genes, responsible for secondary metabolite biosynthesis, showed that VKPM B-3276 has a number of pathogen-related features. Gene clusters for bacillibactin and antrachelin siderophore biosynthesis, system for petrobactin-mediated iron uptake, as well as multiple toxin/antitoxin systems were found. VKPM B-3276 also possesses a heme utilization system, characteristic to gram-positive pathogens [6]. Interestingly, large proportion of pathogen-related gene clusters show a high level of syntheny with extremely pathogenic *B. anthracis* [7], therefore not only accentuating the importance of this strain as a prospective insecticide, but also indicating requirement of extensive safety studies before implementation of this strain in agricultural industry.

**Fig. 2:**
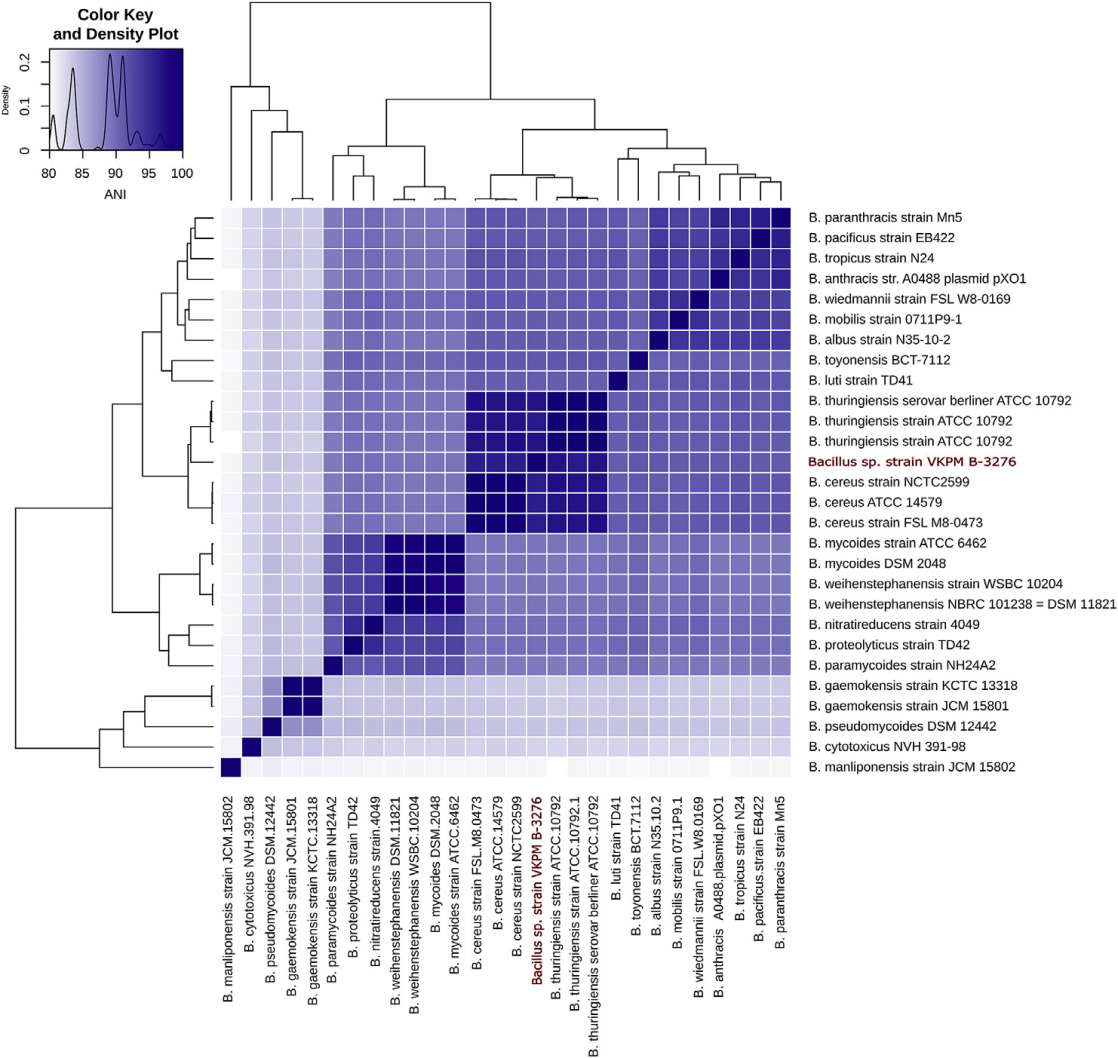
Average nucleotide identity between type strain genome assemblies of “*Bacillus cereus* group”, available at NCBI GenBank, and Bacillus sp. strain VKPM B-3276 (marked in red).

According to the analysis of 16S rRNA genes, strain VKPM B-3276 belongs to “*Bacillus cereus* group” of species, including *B. cereus, B. anthracis* and well known entomocidic strain *Bacillus thuringiensis* [8]. For the purpose of refinement of strain B-3276 phylogenetic position, average nucleotide identity (ANI) was calculated between B-3276 and all available genomes of “*Bacillus cereus* group”. ANI analysis showed that *B. thuringiensis* serovar *berliner* ATCC 10792 (96.48%) and *B. cereus* strain NCTC2599 (95.96% ANI) were the closest relatives of strain VKPM B-3276 forming with it a distinctive cluster (Fig. 2). Digital DNA:DNA Hybridization analysis (DDH) showed that the probability of the hypothesis, that these strains are from the same subspecies, is less than 25%.

## 2 Experimental design, materials and methods

### 2.1 Strain isolation and deposition into collection

VKPM strain B-3276 was isolated from *Culex pipiens* larvae cadaver [2] and deposited in Russian National Collection of Industrial Microorganisms (VKPM). In 2018 it was sequenced in the frame of Russian program “Genomes of industrially-relevant microorganisms”.

### 2.2 DNA extraction, library preparation and sequencing

Genomic DNA was extracted and purified with standard phenol-chloroform method. DNA integrity was assessed by electrophoresis in agarose gel. Fragmentation of DNA was performed with Bioruptor™ sonicator (Diagenode, Belgium) to achieve an average fragment length of 500 bp. Additional step of size-selection with electrophoresis was performed before library preparation to get fragments in range from 400 to 600 bp. Further steps of library preparation were performed with KAPA™ HyperPlus fragment library kit (Roche) according to the manufacturer's instructions. Sequencing was done with Illumina MiSeq™ platform (Illumina, USA) using 500 cycles paired-end sequencing cartridge. 579,166 read pairs were obtained from the sequencing run.

### 2.3 *De novo* assembly

Removal of low-quality reads, bases and sequencing adapters was made with fastq-mcf [9] using the following parameters: Phred score ≥ 25, window size = 5. Genome were assembled with SPAdes v 3.10 [10] in “careful” mode. To check the quality of the assembly, reads were mapped back to contigs with bowtie2 [11], mapping file was processed with samtools [12].

### 2.4 Genome annotation and analysis

Genome was annotated with RAST [2] using RASTtk scheme [13]. Functional analysis was performed using the tools embedded in SEED portal [14]. CAZymes [3] prediction was done using the dbCAN meta server [4]. Analysis of genes involved in the biosynthesis of secondary metabolites was made with ANTISMASH [15] server. Average nucleotide identity was calculated using ani.rb script (https://github.com/lmrodriguezr/enveomics). ANI heatmap was plotted using ggplot2 library for R. Probability of being a new species or subspecies was assessed with GGDC 2.1 [16].
